# Probabilistic reanalysis of storm surge extremes in Europe

**DOI:** 10.1073/pnas.1913049117

**Published:** 2020-01-13

**Authors:** Francisco M. Calafat, Marta Marcos

**Affiliations:** ^a^Department of Marine Physics and Ocean Climate, National Oceanography Centre, Liverpool L3 5DA, United Kingdom;; ^b^Department of Oceanography and Global Change, Mediterranean Institute for Advanced Studies, Spanish National Research Council and University of the Balearic Islands (CSIC-UIB), Esporles 07190, Spain;; ^c^Department of Physics, University of the Balearic Islands, Palma 07122, Spain

**Keywords:** sea level, extremes, storm surge, flooding, Bayesian hierarchical model

## Abstract

Extreme sea levels are a significant threat to life, property, and the environment. These threats are managed by coastal planers through the implementation of risk mitigation strategies. Central to such strategies is knowledge of extreme event probabilities. Typically, these probabilities are estimated by fitting a suitable distribution to the observed extreme data. Estimates, however, are often uncertain due to the small number of extreme events in the tide gauge record and are only available at gauged locations. This restricts our ability to implement cost-effective mitigation. A remarkable fact about sea-level extremes is the existence of spatial dependences, yet the vast majority of studies to date have analyzed extremes on a site-by-site basis. Here we demonstrate that spatial dependences can be exploited to address the limitations posed by the spatiotemporal sparseness of the observational record. We achieve this by pooling all of the tide gauge data together through a Bayesian hierarchical model that describes how the distribution of surge extremes varies in time and space. Our approach has two highly desirable advantages: 1) it enables sharing of information across data sites, with a consequent drastic reduction in estimation uncertainty; 2) it permits interpolation of both the extreme values and the extreme distribution parameters at any arbitrary ungauged location. Using our model, we produce an observation-based probabilistic reanalysis of surge extremes covering the entire Atlantic and North Sea coasts of Europe for the period 1960–2013.

## Significance

Occurrence probabilities of extreme sea-level events are required in the design of flood protection measures. Estimation of these probabilities, however, is challenging due to the small sample of extreme events in the historical sea-level record. We address this challenge by exploiting spatial dependences in the extreme data through a spatiotemporal probabilistic model. Our approach leads to estimates of event probabilities with high accuracy and precision, allows for estimation at ungauged locations, and involves a comprehensive treatment of uncertainties. These three properties make the reanalysis presented here a valuable tool to support both planning decisions in relation to coastal flooding and current efforts to understand the link between extreme events and climate change.

Extreme sea levels can have profound impacts on coastal areas, including significant loss of life and damage to property and to the environment. They cause billion-dollar disaster events in countries across the globe and floods related to sea level now cost the world 10s of billions of dollars each year (1). And with climate projections indicating a significant increase in the intensity and frequency of sea-level extremes by 2100 (2, 3), those numbers are bound to grow even further. To manage these threats, coastal planners use measures of extreme event likelihood to estimate risk and determine appropriate levels of protection that balance expected damage with protection costs. When risk can be accurately estimated, a well-designed risk mitigation plan can save both lives and money by reducing disaster impact while avoiding needless costly overprotection measures. In reality, however, estimates of event probabilities, which are central to risk estimation, are often subject to large uncertainty, primarily due to the sparseness of the observational record. This uncertainty can lead to a significant shortfall in the performance of risk mitigation strategies, including the premature failure of infrastructure, with disastrously expensive consequences. Reducing uncertainty in existing estimates of probabilities of sea-level extremes is therefore a priority, in order to enable more accurate risk estimation, and thus more effective mitigation.

Extreme value theory (EVT) provides the most rigorous statistical framework for the analysis of extremes and underlies the majority of existing work on sea-level extremes. Generally, there are two ways of defining extremes, both widely used: the block-maxima method, which divides the observations into consecutive nonoverlapping blocks (or periods), typically years, and selects the maximum value in each block; and the peaks-over-threshold method, which considers all of the values above a certain threshold. The central result of EVT asserts that the only possible limiting distribution of block maxima is the generalized extreme value (GEV) distribution (4, 5). Analogously, threshold excesses can only converge in distribution to the generalized Pareto distribution (6). This fundamental result means that, assuming the observations are independent and identically distributed, the distribution of extremes can be characterized simply by fitting one of those limiting distributions to the extreme data.

While simple conceptually, the application of EVT to sea-level data involves a number of challenges. First, the historical sea-level record, consisting of tide gauge measurements, provides only a small sample of extreme events, from which accurate estimation of the distribution parameters is difficult. Second, there is abundant observational evidence that the sea-level distribution is changing with time, both its mean values (7) and its tail behavior (8–11), which violates the assumption of stationarity on which classical EVT is predicated. Taking this nonstationarity into account is essential to ensure not only that EVT remains applicable, but also that risk mitigation strategies select a level of protection that matches the real risk of extremes. Last but not least, tide gauge observations are only available at a small number of sites, while estimates of event probabilities are also needed at many other ungauged coastal locations. Dynamical models can provide simulated data with better spatial coverage, but extreme values tend to be underestimated in such models (12–15). Furthermore, existing dynamical simulations span only the most recent decades, and hence they face the same problem of a small sample of events as the observations.

Studies of extremes have become a preeminent focus for sea-level research in recent decades. Although EVT is central to most of these studies, they differ in their geographical scope, their definition of extremes, and in how they address nonstationarity. Some studies are global in scope (8, 9, 14, 16, 17), whereas many others have a regional or local focus (10, 13, 18–28); some studies adopt a block-maxima approach (e.g., ref. 9), while others use threshold excesses (e.g., ref. 23); some incorporate nonstationarity through state-space methods (e.g., ref. 9), whereas others model it using fits to running windows (e.g., ref. 10) or parametric approaches (8). Beyond these differences, all these studies share a common trait: they analyze extremes on a site-by-site basis by fitting a model separately at each tide gauge station. The limitations of this approach are readily apparent. At sites with long tide gauge records, the approach is generally able to constrain the distribution parameters reasonably well, however at sites with little data (the vast majority) estimates have large uncertainty. Crucially, the approach tells us nothing about extremes at ungauged locations.

Here, we present an approach based on a Bayesian hierarchical model (see ref. 29 for a general description of hierarchical models) that addresses the challenges posed by the sparseness of the observational record and overcomes the limitations of the traditional site-by-site analysis. Our focus here is on the surge contribution, which is the part of sea level that remains after removal of the tide and the mean sea level. While spatial hierarchical models have never been used for sea-level extremes (to our knowledge), they have been successfully applied to other types of extremes (30–35), and are emerging as the state-of-the-art tool for geostatistical modeling in general. Our approach relies on the fact that, although observations are spatially discrete, the storm surge process is continuous in space and varies smoothly with length scales similar to those of the weather regimes that give rise to the surges. This means that, although tide gauge sites might have some degree of individuality due, for example, to local bathymetric features, there will also be dependences among them. The key idea is to exploit these dependences in order to enable sharing of information across stations. Such data pooling cannot only drastically reduce estimation uncertainty, but also allows for the interpolation of the extreme surge field at unobserved locations and times.

The idea of pooling information across space has been applied before to the analysis of surge extremes, primarily through the method of regional frequency analysis (RFA) (36, 37). RFA involves, first, predefining homogeneous spatial regions, then normalizing the extreme data in each region by an index flood measure, and last fitting an extreme value distribution to the pooled normalized data. While RFA represents an improvement over the traditional single-site analysis as it allows for more precise estimates of event probabilities, it has its limitations. In particular, the specification of homogeneous regions introduces artificial boundaries that can lead to discontinuities in the extreme field, contradicting the physical expectation that the surge process is spatially continuous. In addition to this, RFA does not permit incorporation of physical information through covariates, and accounting for nonstationarity is unfeasible, or at best problematic. Notably, it is unclear how errors propagate through the various steps of the procedure, which presents an obstacle to obtaining proper uncertainty estimates. Relatedly, RFA typically ignores spatial dependence when estimating the extreme distribution parameters, which artificially narrows confidence intervals, although a modification to correct for this has recently been proposed (38).

In contrast to RFA, our approach captures spatial dependence in both the surge annual maxima, via a max-stable process, and in the GEV parameters, through latent processes and physical covariates. A max-stable process is the infinite-dimensional generalization of the GEV distribution (39), and hence the most appropriate choice to model pointwise maxima. The use of spatial models avoids spatial discontinuities and enables us to flexibly account for site differences while also pooling information across sites. Furthermore, the hierarchical approach allows us to prescribe rich classes of spatiotemporal models. For example, our model introduces temporal variation in the GEV location parameter to account for long-term changes in extremes arising from climate change. Importantly, our approach naturally accommodates data gaps, involves a comprehensive treatment of uncertainties with rigorous error propagation, and allows estimation of both the annual maxima and the GEV parameters at any arbitrary location, either gauged or ungauged. This enables us to produce observation-based estimates of the GEV parameters and annual maxima on a grid covering the entire Atlantic and North Sea coastlines of Europe for the period 1960 through 2013 (40).

This study focuses on describing the Bayesian hierarchical model, assessing its performance through a number of evaluation metrics, and presenting the reanalysis.

## Formulation of the Bayesian Hierarchical Model

A Bayesian hierarchical model is a full probability model that makes inferences from data about unobserved quantities and is expressed, by Bayes’ rule, as a product of conditional distributions or submodels (29, 41): 1) an observation model (called the likelihood) that links the observed data to the spatiotemporal processes; 2) a process model that describes the dynamics of the processes; and 3) a parameter model that models the uncertainty in the parameters and incorporates our prior knowledge about the data and the processes. Bayesian inference relies on evaluating the joint distribution of processes and parameters conditioned on the observed data (called the posterior distribution), and accounts for uncertainty in the observations, processes, and parameters.

In modeling spatial dependence of extremes, it is important to distinguish between two types of dependence (30), which here we refer to as residual and climatological. The former occurs when multiple locations are affected by the same events, while the latter implies locations with similar storminess but not necessarily cooccurrence of events. In other words, residual dependence implies dependence among annual maxima whereas climatological dependence reflects spatial association among the GEV parameters. Here, residual dependence is captured via a max-stable process, while dependence among the GEV parameters is described via latent processes with random effects and bathymetric covariates. The implementation of the max-stable process is the same as described by ref. 32 and we defer to that study for full details.

In our model, the GEV location parameter is allowed to vary in both time and space, whereas the scale parameter varies only in space. The shape parameter is assumed to be constant over the entire domain. This latter assumption is justified by exploratory analysis based on individual GEV fits to the observed annual maxima (*SI Appendix*). Inference in our model is performed using Markov chain Monte Carlo (MCMC) sampling as described in *Materials and Methods*. In the following, we describe in detail the observation and process layers of the hierarchical model. The parameter layer is described in *SI Appendix*.

## Observation Layer

Let *Y*_*t*_(**s**), *t* = 1, …, *T*, be the annual maximum surge for year *t* and at location **s**, and let **s**_1_, …, **s**_*n*_ denote the locations of the tide gauge stations. We assume that the surge process *Y*_*t*_(**s**) is max-stable, and hence its marginal distributions are GEV(*μ*_*t*_(**s**),*σ*(**s**),*ξ*), where *μ*_*t*_(**s**) is the location parameter, *σ*(**s**) is the scale parameter, and *ξ* is the shape parameter. Following ref. 32, the likelihood can be written as:

$$Y_{t}\left(s_{i}\right)|\theta_{t}\left(s_{i}\right),\mu_{t}\left(s_{i}\right),\sigma \left(s_{i}\right),\xi ,\alpha \;\overset{\text{ind}}{∼}\;\text{GEV}\left(\mu_{t}^{*}\left(s_{i}\right),\sigma_{t}^{*}\left(s_{i}\right),\alpha \xi \right),$$ [1]

$$\mu_{t}^{*}\left(s\right)=\mu_{t}\left(s\right)+\frac{\sigma \left(s\right)}{\xi }\left(\theta_{t}{\left(s\right)}^{\xi }-1\right),$$ [2]

$$\sigma_{t}^{*}\left(s\right)=\alpha \sigma \left(s\right)\theta_{t}{\left(s\right)}^{\xi },$$ [3]

where $\theta_{t}\left(s\right)$ is a spatial process capturing residual dependence and α∈(0,1) is a parameter that controls the relative contribution of small-scale errors.

At this point, it is instructive to make a few observations on the three equations above. First, Eq. **1** means that the annual maxima are independent, conditioned on $\theta_{t}\left(s\right)$, $\mu_{t}\left(s\right)$, σ(s), ξ, and α, and are modeled as 1-dimensional GEVs. Second, climatological dependence is captured by specifying spatial models for $\mu_{t}\left(s\right)$ and σ(s), whereas residual dependence is modeled by introducing random effects in the GEV parameters through $\theta_{t}\left(s\right)$, as represented by $\mu_{t}^{*}\left(s\right)$ and $\sigma_{t}^{*}\left(s\right)$. Hence, it is $\theta_{t}\left(s\right)$ that confers the model its ability to make predictions of the annual maxima at ungauged locations. To illustrate how $\theta_{t}\left(s\right)$ induces residual dependence, it is helpful to consider the cases where α→0 and α→1. In the first case, $\sigma_{t}^{*}\left(s\right)\to 0$ and $Y_{t}\left(s\right)$ convergences in distribution to $\text{GEV}\left(\mu_{t}^{*}\left(s\right),0,0\right)$, which implies $Y_{t}\left(s\right)\approx \mu_{t}^{*}\left(s\right)$, and thus the surge process becomes a spatial process with strong residual dependence. Conversely, when α→1, $\theta_{t}\left(s\right)\to 1$ and $Y_{t}\left(s\right)$ convergences to $\text{GEV}\left(\mu_{t}\left(s\right),\sigma \left(s\right),\xi \right)$, and thus there is no residual dependence. Next, we describe how the processes $\theta_{t}\left(s\right)$, $\mu_{t}\left(s\right)$, σ(s) are modeled.

## Process Layer

The spatial residual process $\theta_{t}\left(s\right)$ is expressed as

$$\theta_{t}\left(s\right)={\left(\sum_{l=1}^{L}A_{t,l}w_{l}{\left(s\right)}^{1/\alpha }\right)}^{\alpha },$$ [4]

where $w_{l}\left(s\right)\geq 0$ are kernel functions and $A_{t,l}$ are their coefficients. The kernel functions are taken to be scaled Gaussian functions (other kernels are possible):

$$w_{l}\left(s\right)=\frac{K\left(s|v_{l},\tau \right)}{\sum_{j=1}^{L}K\left(s|v_{j},\tau \right)},$$ [5]

$$K\left(s|v_{l},\tau \right)=\frac{1}{2\pi \tau^{2}}\text{exp}\left(-\frac{1}{2\tau^{2}}{\left(s-v_{l}\right)}^{T}\left(s-v_{l}\right)\right),$$ [6]

where **v**_1_, …, **v**_*L*_ are spatial knots and *τ* is the characteristic length scale of the residual dependence. The scaling ensures that the kernels sum to 1 at each location, which is required to preserve the max-stability properties of the model (see ref. 32). The location of the spatial knots is shown in *SI Appendix*, Fig. S1*A*.

The coefficients of the kernel functions are assumed to follow a positive stable distribution to ensure max-stability (32):

$$A_{t,l}\;\overset{\text{iid}}{∼}\;PS\left(\alpha \right).$$ [7]

The location parameter $\mu_{t}\left(s\right)$ is assumed to vary smoothly with time (i.e., we aim to capture long-term changes such as nonlinear trends as opposed to shorter-term variations which are likely unresolvable), thus we model it as a spatiotemporal integrated random walk of the form

$$\mu_{t}\left(s\right)=\mu_{t-1}\left(s\right)+\mu_{trend,t-1}\left(s\right),$$ [8]

$$\mu_{trend,t}\left(s\right)=\mu_{trend,t-1}\left(s\right)+\omega_{t}\left(s\right),$$ [9]

where $\omega_{t}\left(s\right)$ is a zero-mean Gaussian process (42) $\omega_{t}\left(s\right)∼\text{GP}\left(0,\text{c}\left(s,s';\gamma_{\mu },\rho_{\mu }\right)\right)$, with c(⋅,⋅) being a covariance function and $\gamma_{\mu }$ and $\rho_{\mu }$ denoting, respectively, the SD and length scale defining the covariance function. The initial states of the location parameter and its trend are modeled as

$$\mu_{t=0}\left(s\right)∼\text{GP}\left(x^{T}\left(s\right)\beta_{\mu },\text{c}\left(s,s';\gamma_{\mu_{0}},\rho_{\mu_{0}}\right)\right),$$ [10]

$$\mu_{trend,t=0}\left(s\right)∼\text{GP}\left(0,\text{c}\left(s,s';\gamma_{\mu_{00}},\rho_{\mu_{00}}\right)\right),$$ [11]

where x(s) is a 2 × 1 vector of referenced covariates containing an intercept and the width of the continental shelf at each location, and $\beta_{\mu }$ is a 2 × 1 vector of regression coefficients. The reason for using shelf width as a covariate is justified by the theory of storm surges, which states that wider continental shelves lead to larger storm surges (43).

Finally, the logarithm of the scale parameter, logσ(s), is modeled as a spatial process:

$$\text{log}\sigma \left(s\right)∼\text{GP}\left(x^{T}\left(s\right)\beta_{\sigma },\text{c}\left(s,s';\gamma_{\sigma },\rho_{\sigma }\right)\right).$$ [12]

The scale parameter σ(s) is restricted to be positive, and taking its logarithm ensures that such bound is enforced. This transformation is standard in Bayesian statistics.

For the covariance function c(⋅,⋅) of all Gaussian processes, we assume a Matérn kernel with smoothing parameter ν=5/2 (42).

## Model Validation

Checking that the model performs as expected is a crucial step to ensure that we can confidently use its outputs to learn about extremes. In this regard, we note that, aside from through parameters and processes, uncertainty can enter the model primarily through model inadequacy and lack of observations. It is important to consider these two sources of uncertainty. To this aim, we perform two validation experiments. First, we test the model on synthetic data generated under the same spatiotemporal model as the one used to fit the data. This allows us to isolate the influence from the sparseness of the observational record, since model inadequacy is eliminated by design. The second experiment involves testing the model on real tide gauge data and aims to quantify the real-world skill of the model, but it also allows us to assess, by comparison with the first experiment, the adequacy of the model in reality. For completeness, we perform an additional third experiment with reanalysis data from a dynamical surge model (see *Materials and Methods* for a description of the reanalysis). The surge reanalysis allows us to validate the model at more locations than it is possible using the tide gauge data and provides a means for further assessing the adequacy of the model. The results from the first and third experiments are presented in *SI Appendix*, whereas those from the validation with real tide gauge data are exposed in the following.

A few clarifications first. A description of the tide gauge dataset is given in *Materials and Methods*, while site locations are shown in *SI Appendix*, Fig. S1. Note that whenever we talk about estimates from the hierarchical model and their SEs we are referring to posterior distribution means and SDs, respectively. Model skill is evaluated in terms of a number of diagnostics such as fractional differences (FDs), SEs, and Spearman’s rank correlation for annual maxima predictions (see *Materials and Methods* for an explanation of these diagnostics).

The experiment with synthetic data (*SI Appendix*) shows that, given a perfectly adequate model, the hierarchical model is capable of characterizing the extreme value field at ungauged locations with high accuracy (median FDs of 0.09 and 0.10 for μ and σ, respectively, and a mean Spearman’s rank correlation of 0.70 for annual maxima), despite the sparseness of the tide gauge record. To account for model inadequacy and quantify the real-world skill of the model, we perform the following experiment with real tide gauge data. We exclude one tide gauge site, then predict the extreme value field at the location of the omitted site using the remaining data sites, and compare this prediction with the true values. This procedure is repeated for each one of the 79 tide gauge sites. Since the true value of the GEV parameters is unknown, the predictions of μ and σ at the omitted sites are compared with the Bayesian estimates based on the full dataset. The predicted annual maxima are compared with the actual observed values.

**Fig. 1.**
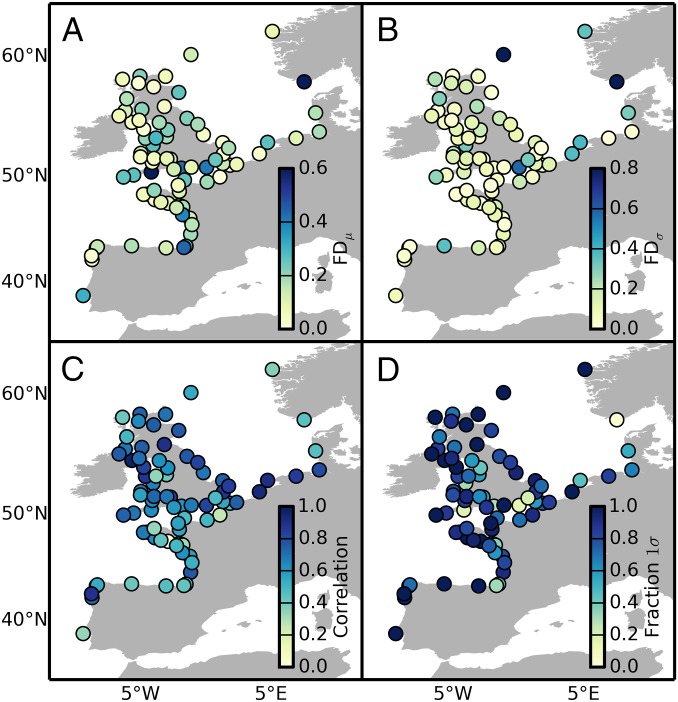
Validation with real tide gauge data. FDs between the Bayesian estimates based on the full tide gauge dataset and the predicted values of the GEV time-mean location (*A*) and scale (*B*) parameters at omitted sites. The Spearman’s rank correlation between the true and predicted annual maxima (*C*), along with the fraction of 1-sigma credible intervals that contains the true extreme value (*D*), are also shown.

Both μ and σ are well captured by the hierarchical model at most excluded data sites (Fig. 1 *A* and *B*), with median FDs of 0.14 and 0.11, respectively. This implies that the estimates of μ and σ at ungauged locations are accurate, on average, to within ∼14 and ∼11% of the “true” value, respectively. Notably, the FDs are fairly uniform across stations, indicating that model skill is largely independent of location. The predictive skill for annual maxima is also good (Fig. 1 *C* and *D*), with a mean Spearman’s rank correlation of 0.62 and a fraction of annual maxima contained by the 1-sigma credible interval of 0.77 (mean value over the 79 stations). The latter is consistent with the theoretical expectation that a 1-sigma credible interval should contain the true value with a probability of ∼0.7, and demonstrates that the hierarchical model yields realistic uncertainty estimates. This performance is similar to that estimated using synthetic data in a perfect model setting (*SI Appendix*), corroborating the adequacy of the hierarchical model. It is also comparable to the results from the validation with reanalysis data (*SI Appendix*), giving us additional confidence in the skill of the model.

## Probabilistic Reanalysis of Storm Surge Extremes

We now present the probability distribution of surge extremes as estimated by the Bayesian hierarchical model from the real tide gauge data. The posterior distribution for the model parameters (α, τ, ξ, $\rho_{\sigma }$, $\rho_{\mu_{0}}$, $\gamma_{\sigma }$, $\gamma_{\mu_{0}}$, $\beta_{\sigma }$, and $\beta_{\mu }$) is shown in *SI Appendix*, Fig. S2. Next we discuss the estimates of the GEV parameters. The values of the time-mean location parameter μ at gauged locations range from 0.4 m along the coastlines of Portugal and Spain to 2 m in the German Bight (*SI Appendix*, Fig. S3*A*). The scale parameter σ shows a similar spatial structure, with values <0.1 m along southwestern Europe and values as large as 0.5 m in the German Bight (*SI Appendix*, Fig. S3*B*). The SEs associated with μ and σ also vary with location (*SI Appendix*, Fig. S3 *C* and *D*) but, in all cases, are at least an order of magnitude smaller than the parameter values (mean values of 2.9 and 1.9 cm, respectively), indicating that the model is able to estimate the parameters at data sites with high precision. The spatial dependence structure underlying the location and scale parameters emerges even more clearly when looking at the gridded estimates (*SI Appendix*, Fig. S4). Both parameters show larger values in the North Sea, particularly in the German Bight, and a general tendency for smaller values as we move southward. SEs at interpolation sites are larger than at gauged locations (mean values of 18.8 cm for μ and 2.5 cm for σ), but they are still only a small fraction of the parameter values at most locations. In particular, SEs are, on average, only 20% of the parameter value, and smaller than 66% (μ) and 46% (σ) of the value at all locations.

**Fig. 2.**
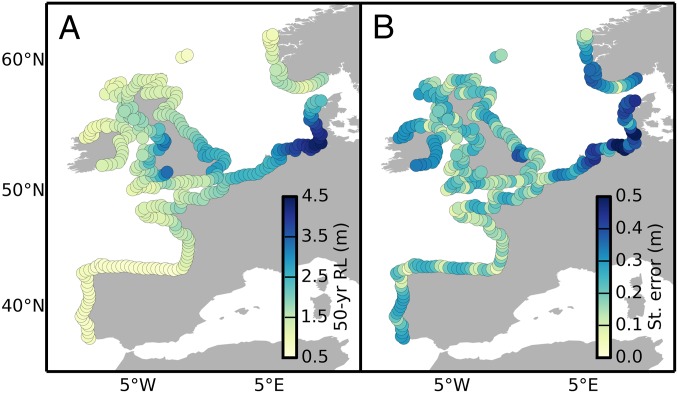
Bayesian estimates of 50-y return levels. Gridded estimates of the 50-y return levels from the hierarchical model (*A*), along with their SEs (*B*). The time-mean value of the location parameter has been used.

The estimates of the GEV parameters can be used to compute extreme event probabilities. In engineering design, such probabilities are typically specified in terms of the *N*-year return level, which is the level that is exceeded on average once every *N* years. The *N*-year return level can be calculated simply as Q(1−1/N;μ,σ,ξ), where Q is the quantile function for the GEV distribution. As an illustration, we show gridded estimates of the time-mean 50-y return level for the European coastlines (Fig. 2). As expected, the smallest return levels are found along the coastlines of Portugal and Spain with values of about 0.7 m, whereas the largest values are found in the North Sea with values as large as 4.4 m in the German Bight (Fig. 2*A*). The uncertainty associated with the estimated return levels is small relative to the value of the return levels, with SEs that range from about 0.2 m almost anywhere outside the North Sea to about 0.3 to 0.4 m in the North Sea (Fig. 2*B*). Note, however, that these SEs refer to interpolation sites; estimates at gauged locations have even smaller uncertainties with a median SE of 0.1 m. To put these uncertainties into context, we note that a single-site GEV model based on maximum likelihood estimation yields estimates of the 50-y return levels with a median SE of 0.2 m, which is two times larger than the Bayesian errors at gauged locations and comparable to the errors at ungauged sites. Furthermore, the single-site model is unable to yield meaningful estimates at three locations (Ferrol in Spain, Le Crouesty in France, and Moray Firth in Scotland) due to the failure of the maximum likelihood estimator to converge.

**Fig. 3.**
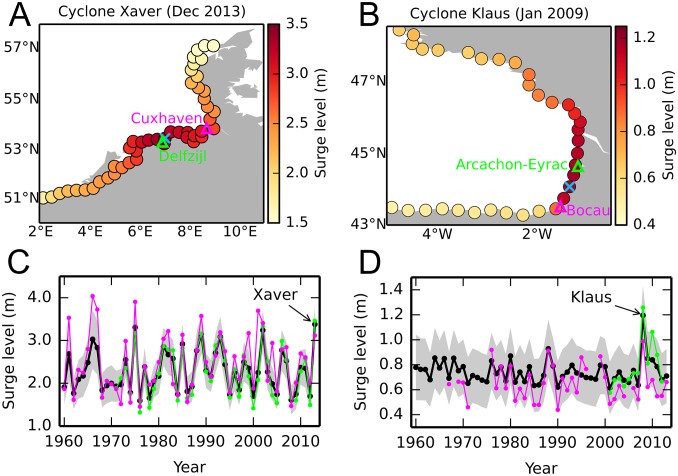
Bayesian predictions of surge annual maxima. Surge levels induced by cyclones (*A*) Xaver in December 2013 and (*B*) Klaus in January 2009 as estimated by the Bayesian hierarchical model. The blue cross denotes the site with the maximum surge whereas the green and magenta triangles denote the location of the two closest tide gauges on either sides of the blue cross. The predicted sequence of annual maxima (thick black line) at the location showing the maximum surge (the blue cross) during (*C*) Xaver and (*D*) Klaus is also shown, along with the observed annual maxima (green and magenta lines) at the two tide gauges shown in *A* and *B*. The gray shading in *C* and *D* denotes the 1-sigma credible interval associated with the predicted annual maxima. Note that years in our analysis start in April, so Cyclone Klaus, which occurred in January 2009, falls into year 2008.

The hierarchical model also produces estimates of the annual maxima in any given year and at any arbitrary location. These estimates enable us, for example, to analyze the magnitude and spatial extent of individual extreme events as they were actually observed. As an illustration, we show the surge levels during the passage of cyclones Xaver in December 2013 and Klaus in January 2009 (Fig. 3), which struck, respectively, the North Sea and Bay of Biscay coastlines and caused record-setting water levels at several coastal locations (e.g., ref. 44). The highest surge levels induced by Xaver occurred near the Germany–Netherlands border (Fig. 3*A*), with values as large as 3.5 m. Surge levels higher than 3 m affected a large section of coastline (>150 km), stretching from northwestern Netherlands to well within the central portion of the German Bight. To put these values into historical context, we also show the predicted time series of annual maxima for the period 1960 through 2013 in the German Bight along with the observed annual maxima at two nearby tide gauges (Fig. 3*C*). We see that, although Xaver is one of the largest events in the period 1960 through 2013, other events of similar magnitude are also evident in both the predicted and observed annual maxima. The series of annual maxima from the two tide gauge records are very coherent between them and also with the predicted annual maxima, giving us confidence in the predictive skill of the model in this region.

Regarding Cyclone Klaus, the maximum surge is found in southwestern France, with values of over 1 m affecting a 300-km section of coastline (Fig. 3*B*). Klaus appears to be the highest surge event over 1960 through 2013 in this region (Fig. 3*D*), both in the predicted and observed sequences of annual maxima. Note, however, that this is a region where the two nearest tide gauges (Arachon-Eyrac and Bocau) show less coherence than those in the North Sea (note, for example, differences in years 2009 and 2010), indicating that smaller length scales might be at work here. They also provide fewer data. The hierarchical model responds to this by widening the credible intervals and pulling the estimate toward the middle of the observed values. This means that events that are localized and show inconsistent values between neighboring stations might be underestimated by the hierarchical model.

## Conclusions and Discussion

Understanding the risk of coastal flooding from sea-level extremes requires accurate estimates of their occurrence probabilities. Obtaining such estimates is, however, challenging because of the difficulties posed by the sparseness of the tide gauge record. There is, therefore, a need for new approaches that can address those difficulties and yield more precise estimates of event probabilities. Here, we have demonstrated that this can be achieved by modeling the surge extreme field as a continuous spatiotemporal process, as opposed to modeling data from each tide gauge station independently. Our approach has enabled us to generate observation-based estimates of event probabilities on a grid covering the entire Atlantic and North Sea coasts of Europe for the period 1960 through 2013. When compared to the traditional single-site modeling approach, our model cuts the uncertainty in estimates of event probabilities (e.g., return levels) by half at gauged locations and yields estimates with comparable uncertainty at ungauged sites. These estimates will help coastal planners and stakeholders to make more confident decisions, particularly in regions with few or no observed data. An additional benefit is that, since our estimates are solely based on observations, they can be used to validate extreme data from dynamical surge models.

Our results are indeed encouraging, but a few remarks need to be made. The ability of the model to share information across space depends directly on the length scales of the extreme field; the smoother the field the more precise the estimates and the further away from a tide gauge we can interpolate with confidence. The GEV parameters tend to vary smoothly in space, following variations in the width of the continental shelf and changes in climate conditions by latitude, and so in general they can be estimated with confidence up to a few hundred kilometers away from any station. We expect this to be the case even in regions affected by tropical cyclones, which have relatively small length scales, since the spatial structure of the GEV parameters is defined by climatological rather than residual dependence. Conversely, coherence among annual maxima is directly related to the spatial extent of individual events and so it tends to show smaller length scales. This means that values of annual maxima at ungauged sites are, in general, more difficult to predict than those of the GEV parameters. The hierarchical model could be extended to capture small-scale effects, for example, by incorporating spatial information via prior distributions, but this would come at the cost of increased model complexity. Other factors that can affect model performance are tide–surge interactions and wave setup effects. The former might introduce errors during the extraction of the surge extremes by causing inaccuracies in the tidal predictions, although here we have aimed to minimize such errors as explained in *SI Appendix*. Wave setup, if present in the tide gauge records after removal of the tide and the mean sea level, might reduce spatial coherence in the extreme values leading to increased uncertainty, but we expect this effect to be small since wave setup is generally small inside harbors where most tide gauges are located. In any case, our model provides realistic uncertainty estimates and these should always be taken into account when using our estimates.

## Materials and Methods

### Tide Gauge Data.

Hourly sea-level observations were obtained from the Global Extreme Sea Level Analysis (GESLA) tide gauge data set (45), which consists of 1,355 records of variable quality and length, obtained from the international databases at the University of Hawaii Sea Level Center and the Global Sea Level Observing System, and complemented with additional observations from national and subnational data providers. The surge annual maxima are extracted from each tide gauge record as described in *SI Appendix*.

### Inference in the Hierarchical Model.

The posterior distribution in our model does not admit a closed-form expression and thus we must resort to MCMC sampling to perform inference. However, inference in our Bayesian hierarchical model is challenging and standard MCMC methods can fail to converge in a reasonable amount of time. Therefore, here we use the No-U-Turn Sampler (NUTS) (46) as implemented by the Stan probabilistic programming language (47). NUTS is a variant of Hamiltonian Monte Carlo with adaptive optimization, and is capable of fitting our model without difficulty, providing fast MCMC mixing and convergence. We run the sampler with four MCMC chains of 2,000 iterations each (warm-up = 1,000). A number of convergence and performance diagnostics for the MCMC sampler are presented in *SI Appendix*.

### Storm Surge Reanalysis from a Dynamical Model.

Daily storm surge maxima from the Global Tide and Surge Reanalysis (GTSR) numerical model (14) along the European coastlines have been used. GTSR provides near-coast time series of storm surges and tides globally at the spatial resolution of the Dynamic Interactive Vulnerability Assessment model (48), spanning the period 1979 through 2014. Storm surge data are simulated with a hydrodynamic model forced with 10-m wind speed and atmospheric pressure from the ERA-Interim global atmospheric reanalysis (49) and run over an unstructured grid with spatial resolution down to 5 km near the coasts.

### Skill Diagnostics.

The skill of the model in estimating the GEV parameters is evaluated in terms of FDs, which are defined as $\text{FD}=\left|\left(x_{true}-\hat{x}\right)/x_{true}\right|$, where $x_{true}$ and $\hat{x}$ are the true and estimated values of the parameter, respectively. Note that, since this metric compares true and estimated values, it provides a measure of the accuracy of the hierarchical model. The SEs yielded by the model, on the other hand, are a metric for model precision.

To estimate the skill of the model in interpolating the annual maxima at ungauged locations, we use two metrics: 1) the Spearman’s rank correlation between the true and predicted extreme values; and 2) the fraction of true extreme values that fall within the 1-sigma credible interval. That is, at each location, we calculate how many of the 54 true extreme values over 1960 through 2013 are contained by the 1-sigma credible intervals, and then divide the result by 54.

### Data and Code Availability.

The probabilistic surge reanalysis data presented in this paper and the code to implement the hierarchical model are both available via Zenodo, DOI: 10.5281/zenodo.3471600; DOI: 10.5281/zenodo.3442167.

## Supplementary Material

Supplementary File
