# Supplementary material for: Probabilistic reanalysis of storm surge extremes in Europe
Source: Proc Natl Acad Sci U S A. 2020 Jan 13;117(4):1877–83. doi: 10.1073/pnas.1913049117 (PMC6994974; doi:10.1073/pnas.1913049117)
Supplement: Supplementary File [file pnas.1913049117.sapp.pdf]

Supplementary Information for

Probabilistic reanalysis of storm surge extremes in Europe

Francisco M. Calafat*, Marta Marcos

*Corresponding author.

E-mail: francisco.calafat@noc.ac.uk

This PDF file includes:

- Supplementary text
- Figures S1 to S12
- Table S1
- SI References

www.pnas.org/cgi/doi/10.1073/pnas.1913049117

Supplementary Information Text

Exploratory analysis on the spatial structure of the GEV shape parameter

Here we demonstrate, based on quantitative analysis, that it is appropriate to model the GEV shape parameter as being spatially constant. We begin by showing the shape parameter values at each tide gauge station as estimated by individual GEV fits (SI Appendix, Fig. S5A). While the map suggests some small regions of coherence, the spatial structure is much weaker than in the case of the location and scale parameters and there are significant differences, even in sign, between nearby stations. As a means of establishing whether the differences in the shape parameter across tide gauges reflect true differences or sampling error, we have conducted the following analysis. First, we simulate data from a GEV with the location and scale parameters set to the actual observed values (estimated using individual GEV fits) at each tide gauge site but with a constant shape parameter for all sites. The sample size of the simulated data at each site is the same as that in the tide gauge record. Then we estimate the value of the shape parameter from the simulated data at each site using the single-site GEV model and compare those with the values derived (also using individual GEV fits) from the observed data. Histograms of the two sets of shape parameters are very similar (SI Appendix, Fig. S5B), suggesting that the differences in the shape parameter are likely due to sampling error (i.e., small sample sizes). Indeed, a two-sample Kolmogorov-Smirnov test (1) indicates that the real and simulated shape parameters are very likely to have the same underlying distribution. These results give us confidence in our decision to treat the shape parameter as spatially constant.

Parameter layer of the Bayesian hierarchical model

Here we adopt a full Bayesian approach, and hence all model parameters are estimated from the observations. Note that, in order to facilitate sampling in our model, some parameters are rescaled so that they are approximately on a unit scale. The following priors are ascribed to the (rescaled, where appropriate) model parameters:

- For the shape parameter ξ we assume a uniform distribution: $\xi \sim \mathcal{U}(-0.3, 0.3)$. The lower and upper bounds are selected based on the results of individual GEV fits to the observed annual maxima.
- The parameter α is bounded to be in the range $(0,1)$, and thus we let $\alpha \sim \mathcal{U}(0,1)$.

- The length scale of the kernel functions, τ, is assigned a half-normal distribution: $\tau \sim \text{half-}\mathcal{N}(0,0.5)$. A standard deviation of 0.5 corresponds to half the synoptic scale (~1000/2 km), which is a measure of the spatial extent of extratropical cyclones.
- For the standard deviations of the Gaussian processes we assume a half-normal distribution: $\gamma_{\mu}, \gamma_{\mu_0}, \gamma_{\mu_{00}}, \gamma_{\sigma} \overset{\text{ind}}{\sim} \text{half-}\mathcal{N}(0,1)$. A half-normal distribution is one of the recommended priors for scale parameters in hierarchical models (2, https://github.com/stan-dev/stan/wiki/Prior-Choice-Recommendations) as it enables us to constrain the value of a parameter from above while allowing it to be arbitrarily close to zero.
- In assigning priors to the length scale parameters of the Gaussian processes, we should note that there is no information in the observed data to characterize scales above the maximum distance between stations. The priors should encode this information, and hence we impose a half-normal distribution: $\rho_{\mu}, \rho_{\mu_0}, \rho_{\mu_{00}}, \rho_{\sigma} \overset{\text{ind}}{\sim} \text{half-}\mathcal{N}(0,0.7)$. A standard deviation of 0.7 corresponds to about one third of the maximum distance between stations.
- For the regression coefficients, $\boldsymbol{\beta}_{\mu}$ and $\boldsymbol{\beta}_{\sigma}$, we assume a normal distribution: $\boldsymbol{\beta}_{\mu}, \boldsymbol{\beta}_{\sigma} \overset{\text{ind}}{\sim} \mathcal{N}(0,1.5)$.

The prior distributions are shown in SI Appendix, Fig. S2.To assess the sensitivity of our results to prior choices, we have compared estimates for the cases where the scale parameters of the Gaussian processes are assigned the following priors: half-$\mathcal{N}(0,1)$, half-$\mathcal{N}(0,2)$, half-$\mathcal{N}(0,10)$. The results are shown in the SI Appendix, Table S1. We note that the estimates of the various scale parameters ($\gamma_{\mu}, \gamma_{\mu_0}, \gamma_{\mu_{00}}, \gamma_{\sigma}$) are fairly consistent across all three cases. Furthermore, differences in the estimates of the GEV location and scale parameters among the three cases are negligible. Our estimates are also fairly insensitive to the choice of priors for all the other parameters. However, as mentioned above, the assignment of priors to the length scales of the Gaussian processes needs careful consideration, especially for ρ_{μ_0} and $\rho_{\mu_{00}}$. In particular, the likelihood for these two parameters can become non-identified if the scale of the half-normal prior is set to a large value.

Validation with simulated data in a perfect model setting

Here we estimate the skill of the model in a perfect model setting. We first simulate a spatiotemporal process under a max-stable model and sample it at exactly the same times and locations as the tide gauge record (see Materials and Methods for a description of the tide gauge data set and SI Appendix, Fig. S1 for site locations and availability through time). Then, we fit our hierarchical model to the synthetic tide gauge data and inspect whether the model is able to adequately characterize the simulated process, at both gauged and ungauged locations. Estimates from the hierarchical model are compared to estimates derived using a single-site GEV model, as a way of establishing a baseline against which to measure the skill of our model. In this experiment, the GEV location and scale parameters (μ and σ) are spatially variable, whereas the shape parameter ξ is kept constant. Note that, since here μ is assumed constant in time, $\mu_{trend,t} = 0$ and thus $\mu_t = \mu_{t=0}$ for all t (see model formulation). The single-site model used here involves fitting a GEV separately at each site using maximum likelihood estimation, which is the most commonly used method. The results presented next are based on a single realization of a max-stable process, but comparable results are found for other realizations.

We first note that the hierarchical model captures the true value of all model parameters (SI Appendix, Fig. S6), including the length scales (ρ_μ, ρ_{μ_0}) and standard deviations ($\gamma_\mu, \gamma_{\mu_0}$) of the Gaussian processes, which are in general weakly identified and difficult to estimate (3). We note that the value of the parameters used in the simulations has been chosen to be similar to that found in the real tide gauge data, and so, assuming the adequacy of the model, we can expect an equivalent performance when analyzing the actual observations.

Next, we evaluate the skill of the hierarchical model in estimating the marginal GEV parameters μ and σ, at gauged locations. Model skill is assessed in terms of fractional differences (FDs) (see Materials and Methods). The standard errors associated with the estimated values are also shown. Estimates of μ based on the hierarchical model are very close to the true value at all gauged locations, as indicated by a median FD of 0.03 (SI Appendix, Fig. S7A). The single-site model also gives a very good match to the true values with a median FD of 0.03 (SI Appendix, Fig. S7B). While both models exhibit very small FDs, the hierarchical model yields much more precise estimates, as indicated

by their smaller standard errors (median of 2.4 cm vs 3.4 cm) (SI Appendix, Fig. S7C and D).

Differences in performance between the two models become even more apparent when looking at the scale parameter σ (SI Appendix, Fig. S8), for which the hierarchical model exhibits smaller FDs (median of 0.08 vs 0.11) and standard errors that are almost half those of the single-site model (median of 1.5 cm vs 2.6 cm). Note also that, while the FDs and standard errors for the hierarchical model are fairly uniform across stations, those for the single-site model show a much larger spread. In particular, there are several stations where FDs for the single-site model are larger than 0.4 and standard errors are more than three times larger than those from the hierarchical model. Furthermore, there are two stations where the single-site model is unable to provide an estimate due to convergence failure, highlighting the difficulty of this model to constrain the GEV parameters at sites with few data.

The hierarchical model has also a good predictive skill in capturing both μ and σ at ungauged sites (SI Appendix, Fig. S9), with median FDs of 0.09 and 0.10 and median standard errors of 14.9 cm and 2.3 cm, respectively. As expected, FDs and standard errors tend to be larger at locations distant from any tide gauge station, but even at such locations the differences between the true and estimated values tend to be much smaller than the value of the parameter, providing confidence in the skill of the model at ungauged sites. In particular, FDs < 0.5 are found at more than 92% of all interpolation sites, for both μ and σ. Note also that FDs and standard errors for σ at ungauged locations are slightly smaller than those for the single-site model at gauged sites.

To estimate the skill of the model in interpolating the annual maxima we use the Spearman's rank correlation between the true and predicted extreme values, and the fraction of true extreme values that fall within the 1-sigma credible interval (see Materials and Methods). The mean Spearman's rank correlation over all prediction sites is 0.70 (SI Appendix, Fig. S10A), indicating a good predictive skill. Furthermore, correlations > 0.5 are found at 96% of the locations, showing that model skill is largely independent of location. We find that the 1-sigma credible interval encompasses the true extreme value, on average, 73% of the times (SI Appendix, Fig. S10B).

Validation with reanalysis data from a dynamical surge model

The results of the experiment with real tide gauge data represent our most accurate assessment of the predictive skill and accuracy of the hierarchical model in the real world. However, such assessment is only possible at gauged locations. A surge reanalysis, though being only an approximate representation of the real world, gives us an opportunity to assess the model at additional locations and allows for a further assessment of the adequacy of the model. Here, we sample the annual maxima from the reanalysis at the same times and locations as the tide gauge record, then fit our hierarchical model to the sampled data and make predictions of the GEV parameters and the annual maxima at ungauged locations where the reanalysis provides data. The predictions of μ and σ are compared with estimates based on individual GEV fits to the full (i.e., no missing values) reanalysis data, whereas the predicted annual maxima are compared with the actual annual maxima from the reanalysis.

The μ and σ parameters are well captured at most locations (SI Appendix, Fig. S11A and B), with median FDs of 0.07 and 0.20, respectively. The FDs show significant uniformity across most sites, but in the case of σ we note increased FD values (~0.7) along the southern coast of England. Additional analysis suggests that these larger values are due to a sharp gradient in the variance of the reanalysis annual maxima across the English Channel (SI Appendix, Fig. S12A), which the hierarchical model is unable to capture. The fact that the actual tide gauge observations do not show such a pronounced gradient (SI Appendix, Fig. S12B) suggests that the gradient might be a model artefact. Regardless, FDs for μ and σ are <0.5 at 98% and 87% of the locations, respectively, which again confirms the high accuracy of the model. For the prediction of the annual maxima, we find a very high mean Spearman's rank correlation of 0.89 and a fraction of annual maxima contained by the 1-sigma credible interval of 0.81 (average value) (SI Appendix, Fig. S11C and D). This correlation is significantly higher than the one found in the validation with real data (0.62). The reason is that residual dependence in the reanalysis is much stronger than in the observations, as indicated by the smaller value of the parameter α in the reanalysis (0.25 vs 0.54).

Extraction of annual maxima from the tide gauge records

Our analysis of extremes is based on surge annual maxima, which are extracted from each tide gauge record as follows. First, it is important to recognize that tide gauge

records often contain datum shifts that, if went unnoticed, could result in anomalous extreme values. To identify and correct jumps in the sea-level records, we use a parametric global method (4) that aims to detect abrupt changes in the mean of a signal. This algorithm is applied to the low-pass filtered (cutoff 36 hours) records subject to the condition that there should be a minimum of 15 days between changepoints. This condition is necessary to avoid falsely identifying changes associated with surges as datum shifts. The identified jumps are then adjusted in the original records by correcting the mean difference at changepoints.

At this point, we note that waves are typically filtered out from tide gauge records, either mechanically or by time averaging of the sea-level records. Wave setup effects are not explicitly removed, however such effects are generally not captured by tide gauges due to their location inside harbors where water is relatively deep (compared to shallow sloping beaches on which wave setup is most important). This means that the part of sea level that remains after removal of the tide and the mean sea level is primarily the storm surge. As a means of removing the mean sea level, the annual medians along with a seasonal cycle are subtracted from the shift-adjusted tide gauge records. Note that this eliminates any influence from sea-level rise and other long-term sea-level variations on the annual maxima. The tidal component is then estimated, on a year-by-year basis, through harmonic analysis using the program t-tide (5) and removed from the time series to obtain the surges. In doing this, we note that sometimes tide-surge interaction and timing errors can cause inaccuracies in the tidal predictions, leading to artificially large surges. This issue is addressed by first computing the instantaneous phase difference between the observed and predicted signals at the frequency of the main tidal constituent using the Hilbert transform, and then shifting (in time space) the tidal prediction by the amount necessary to remove the phase difference. The shifting is applied cycle by cycle and only if the phase difference in the cycle is larger than 30 minutes. We should note that only a small number of extreme events in the final set correspond to cycles where a phase-shift correction has been applied. The hourly residual time series after removal of the mean sea level and the tides are then visually inspected to identify and remove outliers. Finally, we extract the annual maxima from the residual time series, where we should note that years are defined as starting on 1 April and only years with at least 6000 valid hourly values are considered. The final set of extremes consists of 2500 annual maxima from a total of 79 tide gauge records spanning the period 1960-2013 (the location of the tide gauges is shown in SI

Appendix, Fig. S1A). Note that the number of tide gauge sites with available data decreases rapidly as we go backwards in time (SI Appendix, Fig. S1B).

MCMC diagnostics for the Bayesian hierarchical model

Here we validate the fit of the hierarchical model to the real annual maxima data through a number of MCMC diagnostics. This part of the validation aims to assess whether the sampler has converged and provides good mixing. We begin by looking at convergence diagnostics. While, in practice, there is no definitive way to prove convergence, there are a number of diagnostics that allow us to check certain necessary (albeit not sufficient) conditions for convergence. One of such conditions is that, in equilibrium, samples from different Markov chains should all have the same distribution, regardless of the initial values of the chains. The potential scale reduction statistic (6), $\hat{R}$, tests for this by comparing the sample variances both within individual chains and across multiple randomly initialized chains. At convergence $\hat{R}$ should be close to 1 for all parameters in the model, whereas $\hat{R} > 1.1$ is indicative of non-convergence. We find that $\hat{R}$ is below 1.1 for all parameters in our model, suggesting that all four Markov chains have converged to the equilibrium distribution and are providing a good approximation to the posterior distribution.

In addition to the issue of convergence, another difficulty posed by MCMC methods is that they tend to produce highly correlated samples. The higher the autocorrelation the larger the MCMC standard error, and thus the further the posterior mean will be from the true value of the parameters. As a measure of autocorrelation, we use an estimate of the effective sample size, n_{eff}, for each parameter (7). In general, a n_{eff} per iteration < 0.001 is indicative of poorly mixing chains and suggestive of possible biased estimates. In our hierarchical model, we find $n_{eff} > 0.3$ for most parameters, with the parameter α showing the lowest n_{eff} among all parameters with a value of 0.02. These results indicate low autocorrelation and good mixing.

Finally, there are several additional diagnostics specific to Hamiltonian Monte Carlo, such as divergent transitions and maximum tree depth, which can help diagnose problems with the sampler. In particular, the presence of divergences and/or tree-depth saturation indicates that the sampler is not able to fully explore the posterior distribution and the estimates are likely to be biased. Our analysis of these diagnostics shows that there were no divergences in our fit and none of the iterations saturated the maximum tree depth.

All diagnostics reported above indicate that the hierarchical model provides an adequate fit to the observed annual maxima and that our sampler is accurately characterizing the posterior distribution.

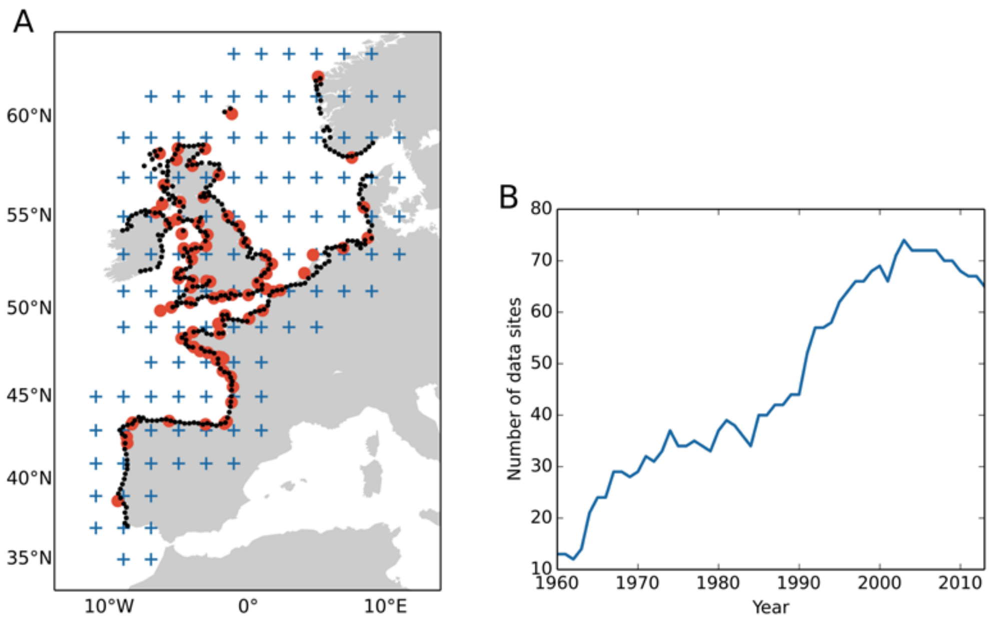

Figure S1. **Availability of the tide gauge data**. (A) Location of the tide gauge stations used in the analysis of extremes (red circles), along with the interpolation grid points (black dots) and the spatial knots used to construct the spatial residual process (blue crosses). (B) The number of tide gauge sites providing data over time (1960-2013).

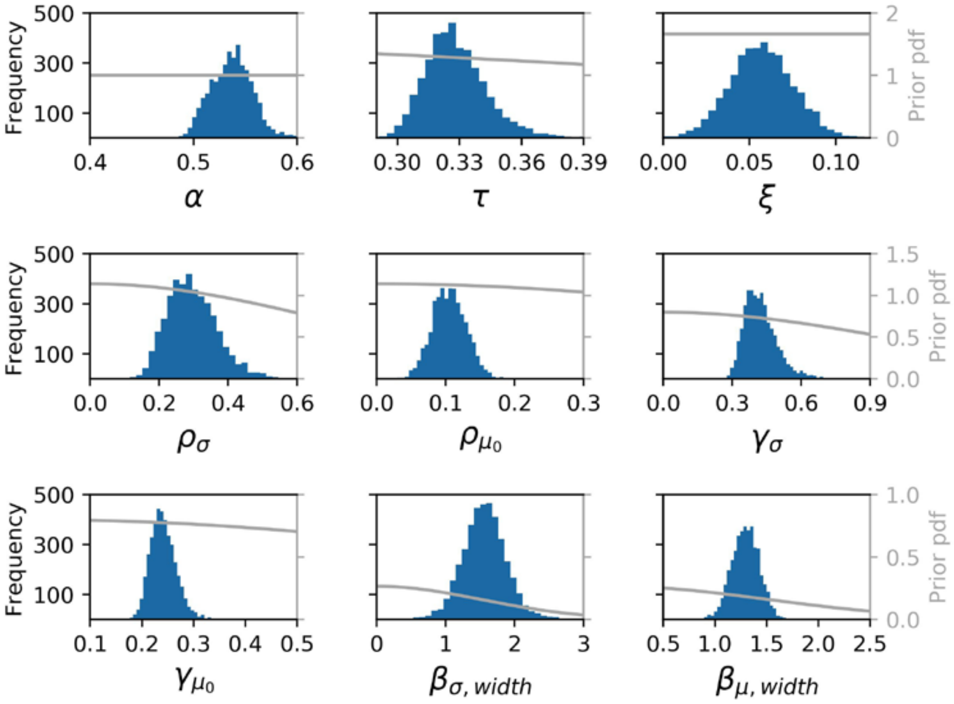

Figure S2. **The posterior distribution for model parameters in the probabilistic reanalysis of storm surge extremes**. Histograms of 4000 draws from the posterior distribution for α, τ, ξ, ρ_{σ}, ρ_{μ_0}, γ_{σ}, γ_{μ_0}, $\beta_{\sigma,width}$ and $\beta_{\mu,width}$ (the subscript *width* denotes regression coefficient associated with the shelf width) as estimated by the Bayesian hierarchical model based on the real tide gauge data. The prior distributions are also shown (gray, right y-axis).

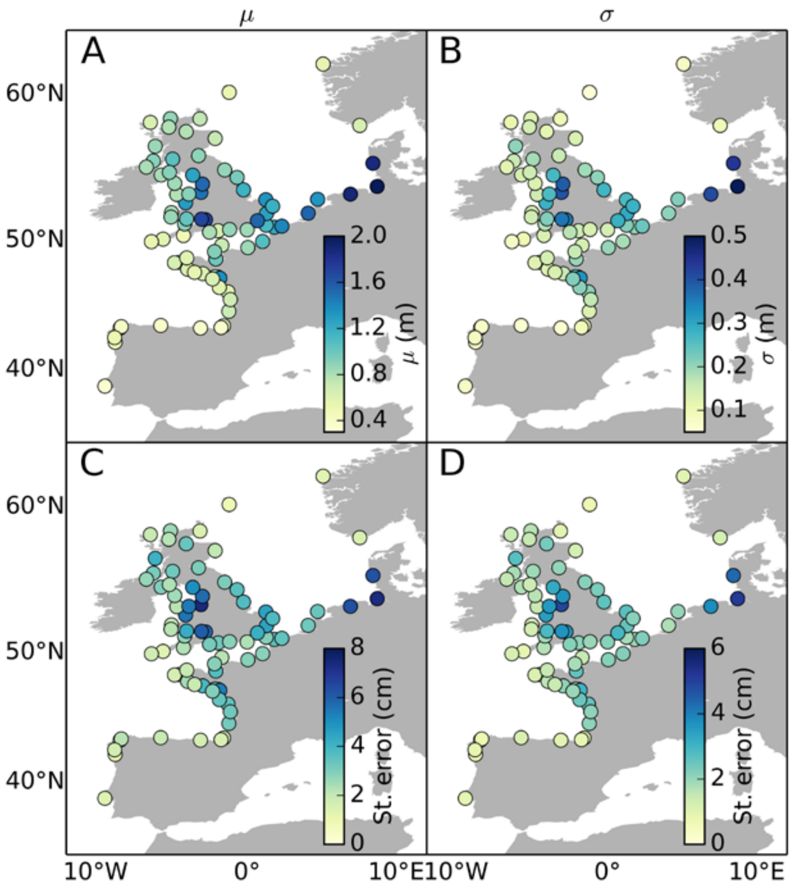

Figure S3. **Bayesian estimates of the GEV parameters from real tide gauge data at gauged locations**. Estimates from the hierarchical model at gauged locations for the GEV time-mean location (A) and scale (B) parameters, along with their standard errors (C and D).

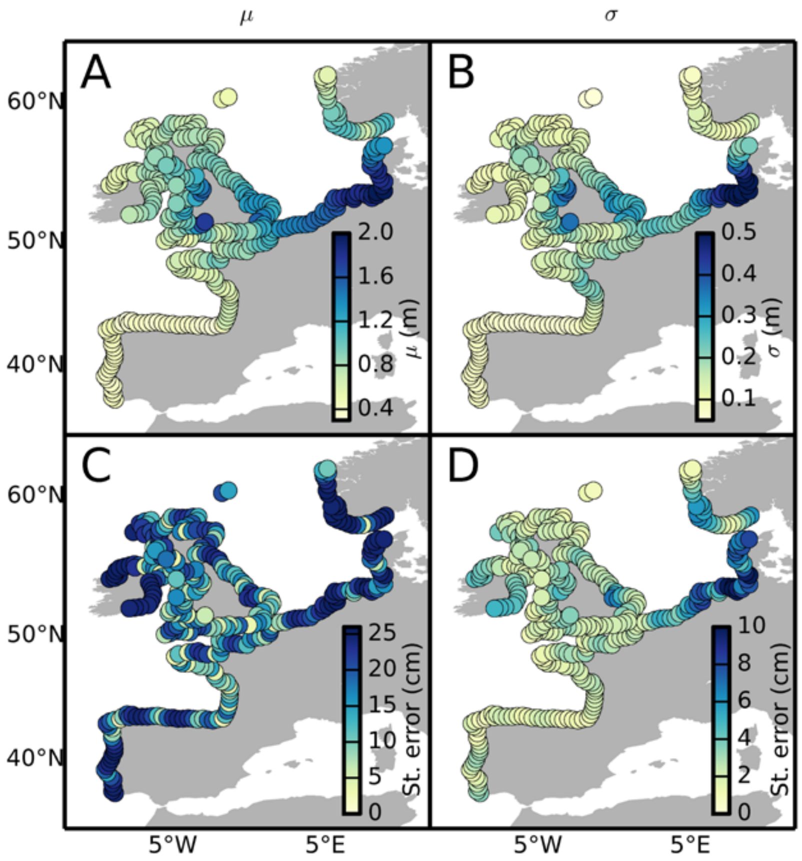

Figure S4. **Bayesian estimates of GEV parameters at ungauged locations**. Gridded estimates of the GEV time-mean location (A) and scale (B) parameters from the hierarchical model, along with their standard errors (C and D).

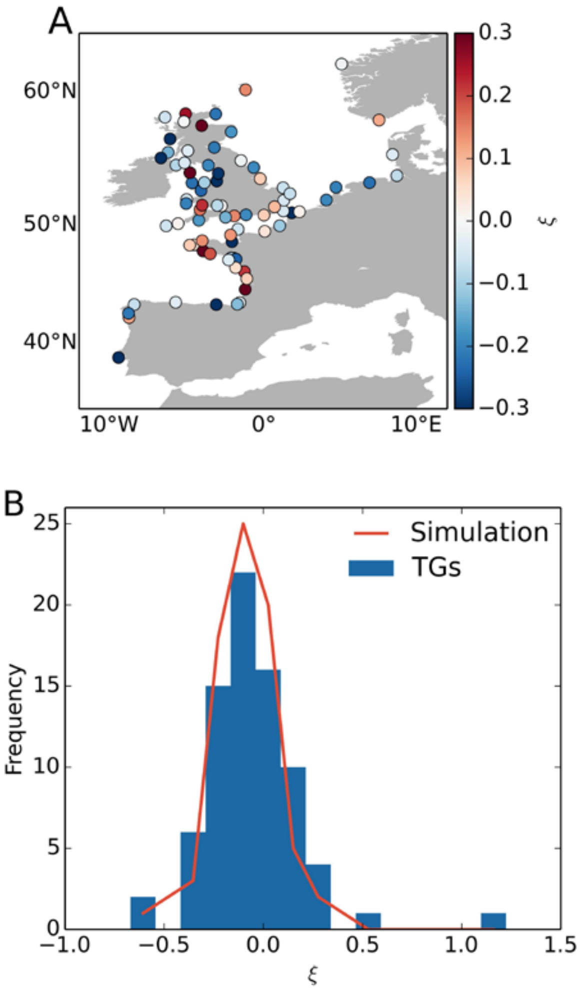

Figure S5. **The spatial structure of the GEV shape parameter**. (A) Estimates of the shape parameter at each tide gauge location based on a single-site GEV model (estimates at two locations have been omitted due to lack of convergence of the maximum likelihood estimator) applied to the observed annual maxima. (B) histogram of the shape parameter estimates shown in panel A, along with the histogram of the shape parameter values derived from simulated data based on a GEV with constant shape parameter.

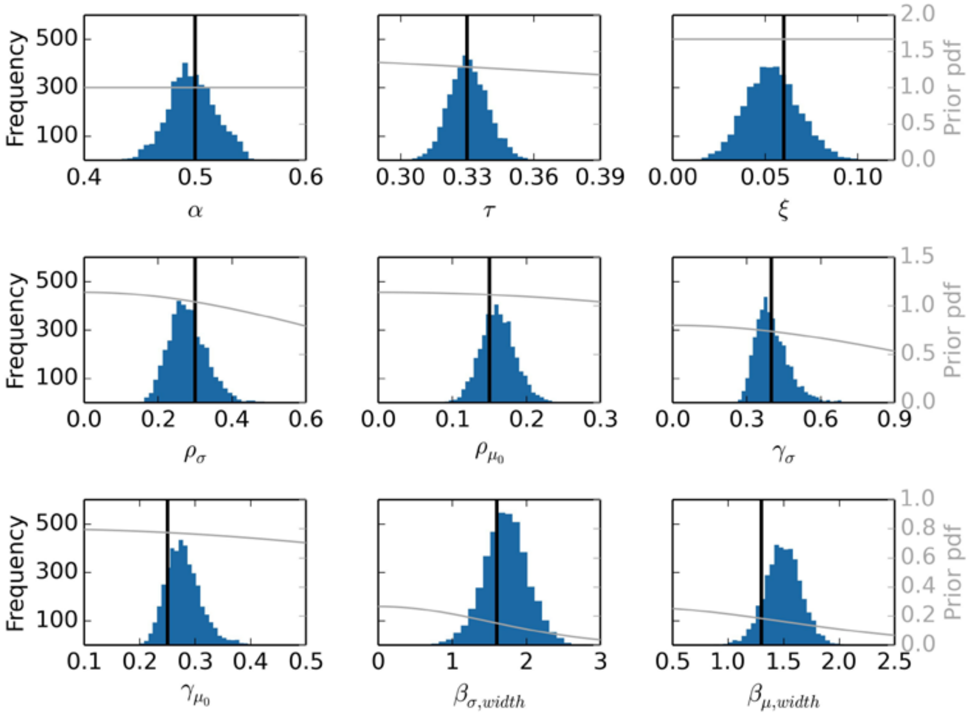

Figure S6. **The posterior distribution for model parameters in the validation with simulated data in a perfect model setting**. Histograms of 4000 draws from the posterior distribution for α, τ, ξ, ρ_σ, ρ_{μ_0}, γ_σ, γ_{μ_0}, $\beta_{\sigma,width}$ and $\beta_{\mu,width}$ (the subscript *width* denotes regression coefficient associated with the shelf width) as estimated by the Bayesian hierarchical model based on simulated data generated under a max-stable model. The vertical black line denotes the true value of the parameters. The prior distributions are also shown (gray, right y-axis).

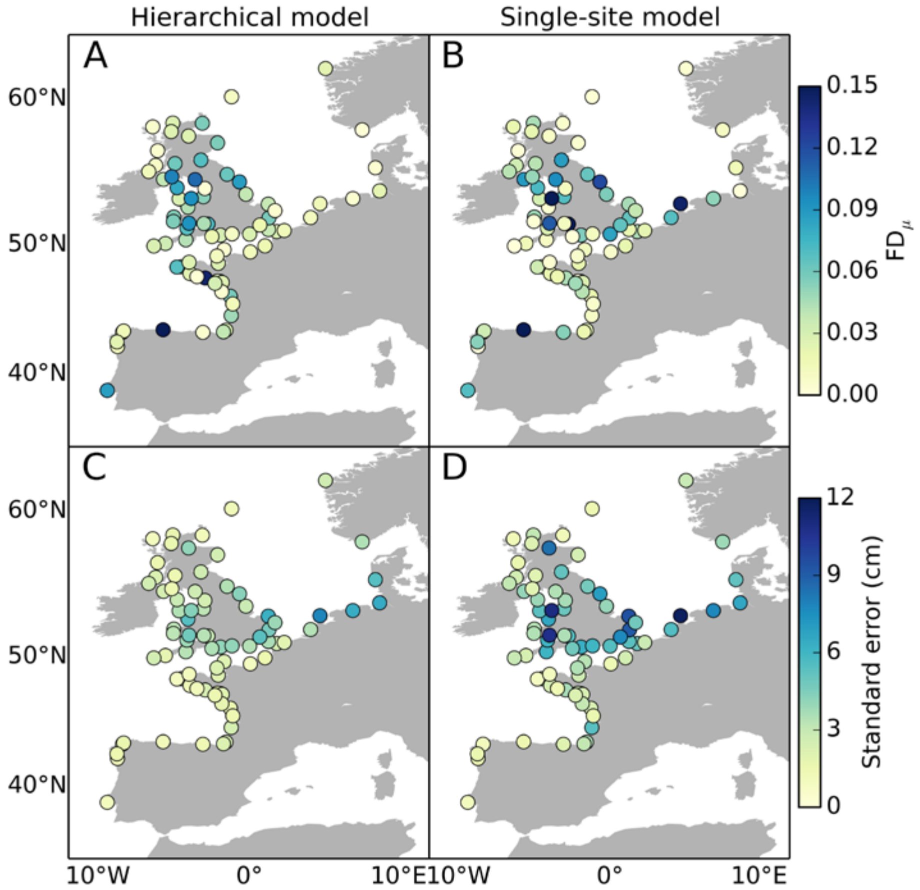

Figure S7. **Validation of the GEV location parameter with simulated data in a perfect model setting at gauged locations**. Fractional differences (FDs) between the true value of the GEV location parameter μ at gauged locations and model estimates from a spatiotemporal hierarchical model (A) and from a single-site GEV model (B), along with the standard errors associated with such estimates (C and D).

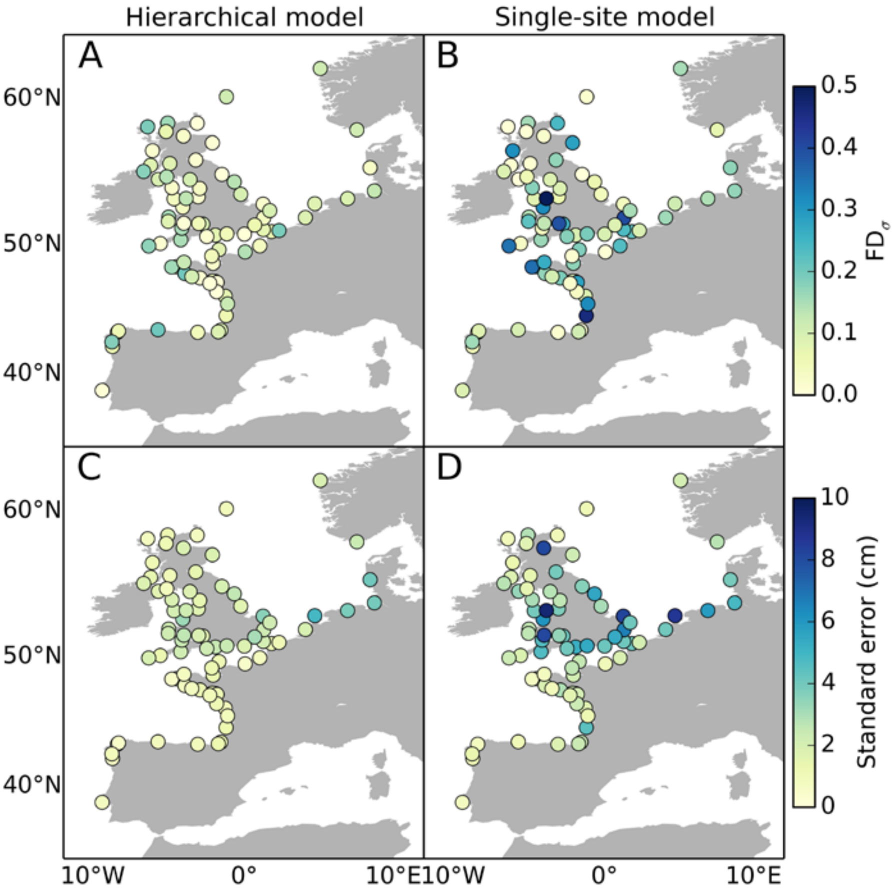

Figure S8. **Validation of the GEV scale parameter with simulated data in a perfect model setting at gauged locations**. Fractional differences (FDs) between the true value of the GEV scale parameter σ at gauged locations and model estimates from a spatiotemporal hierarchical model (A) and from a single-site GEV model (B), along with the standard errors associated with such estimates (C and D).

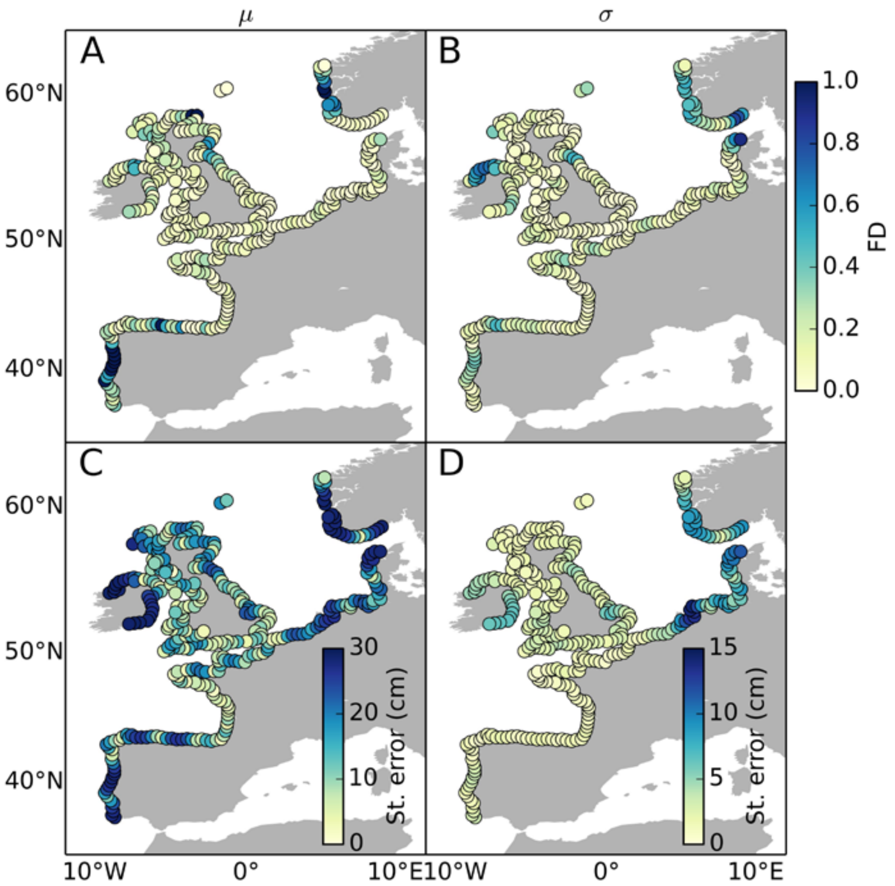

Figure S9. **Validation of the GEV location and scale parameters with simulated data in a perfect model setting at ungauged locations**. Fractional differences (FDs) between the true and estimated values of the GEV location (A) and scale (B) parameters at interpolation locations, along with the standard errors associated with the estimates (C and D).

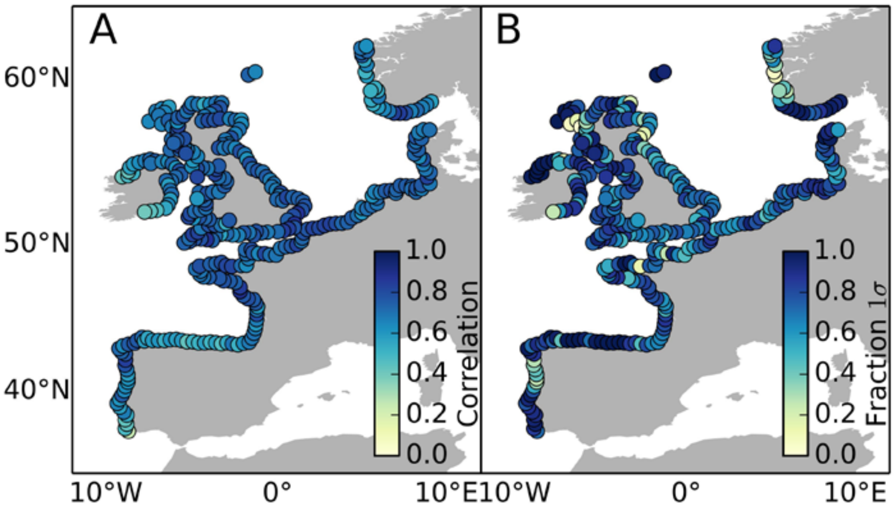

Figure S10. **Validation of the predicted annual maxima with simulated data in a perfect model setting**. Spearman's rank correlation between the true and predicted extreme values at ungauged locations based on the simulated data (A), and the fraction of 1-sigma credible intervals that contain the true extreme value (B).

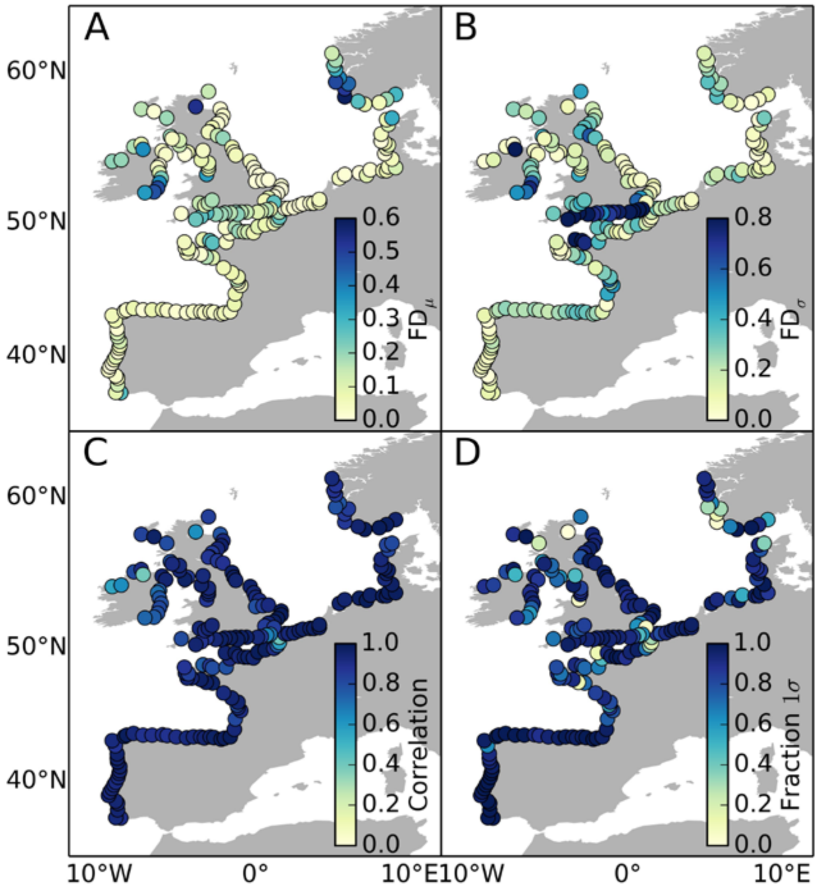

Figure S11. **Validation with reanalysis data from a dynamical surge model**. Fractional differences (FDs) between the true (single-site model estimates based on the full reanalysis data) and the predicted values of the GEV location (A) and scale (B) parameters. The Spearman's rank correlation between the true and predicted annual maxima (C), along with the fraction of 1-sigma credible intervals that contain the true extreme value (D), are also shown.

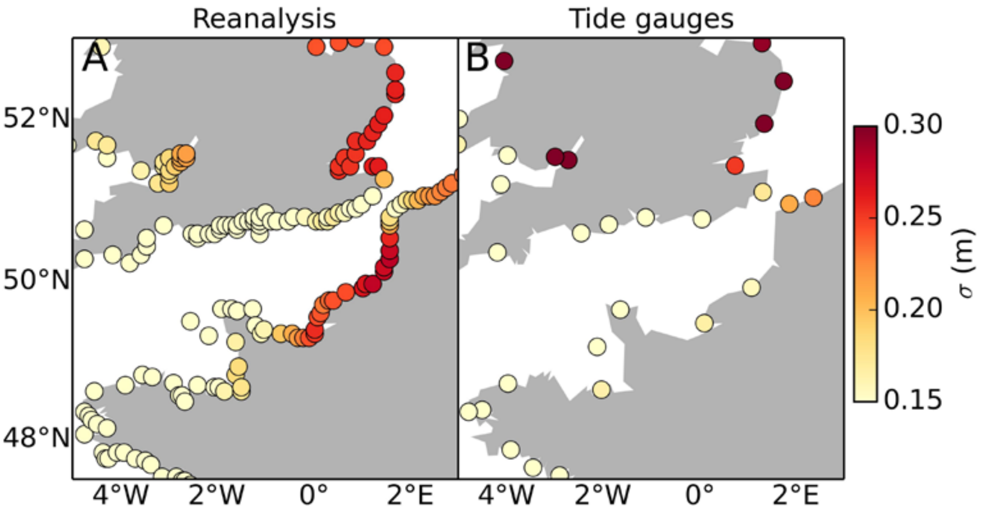

Figure S12. **Sharp gradient of the GEV scale parameter in the dynamical surge reanalysis**. Comparison of the GEV scale parameter at every grid point in the surge reanalysis (A) with that at tide gauge stations (B). The scale parameter values have been estimated by individual GEV fits to the annual maxima.

Table S1. Estimates (mean ± 1-sigma) of the scale parameters of the Gaussian processes ($\gamma_{\mu}, \gamma_{\mu_0}, \gamma_{\mu_{00}}, \gamma_{\sigma}$) under the following prior distributions: half-$\mathcal{N}$(0,1), half-$\mathcal{N}$(0,2), half-$\mathcal{N}$(0,10). The mean difference (over the 79 tide gauge stations) in estimates of the GEV location ($d\mu$) and scale ($d\sigma$) parameters respect to the case with a half-$\mathcal{N}$(0,1) prior is also shown for all three cases.

**Prior**	$\boldsymbol{\gamma_{\mu}}$	$\boldsymbol{\gamma_{\sigma}}$	$\boldsymbol{\gamma_{\mu_0}}$	$\boldsymbol{\gamma_{\mu_{00}}}$	$\boldsymbol{d\mu}$**(%)**	$\boldsymbol{d\sigma}$**(%)**
half-$\mathcal{N}$(0,1)	0.24±0.03	0.43±0.08	0.15±0.11	0.39±0.26	0.00	0.00
half-$\mathcal{N}$(0,2)	0.24±0.03	0.42±0.07	0.16±0.11	0.37±0.27	0.15	0.54
half-$\mathcal{N}$(0,10)	0.24±0.03	0.42±0.07	0.17±0.12	0.45±0.32	0.17	0.77

SI References

1. Massey, F. J., The Kolmogorov-Smirnov Test for Goodness of Fit. J. Am. Stat. Assoc. **46**, 68–78 (1951).
2. A. Gelman, Prior distributions for variance parameters in hierarchical models. *Bayes. Anal.* **1**, 515–533 (2006).
3. H. Zhang, Inconsistent estimation and asymptotically equal interpolations in model-based geostatistics. *J. Am. Stat. Assoc.* **99**(465), 250–261 (2004).
4. R. Killick, P. Fearnhead, I. A. Eckley, Optimal detection of changepoints with a linear computational cost. *J. Am. Stat. Assoc.* **107**, 1590–1598 (2012).
5. R. Pawlowicz, B. Beardsley, S. Lentz, Classical tidal Harmonic Analysis Including Error Estimates in MATLAB using T_TIDE. *Comput. Geosci.* **28**, 929–937 (2002).
6. A. Gelman, D. B. Rubin, Inference from Iterative Simulation Using Multiple Sequences. *Stat. Sci.* **7** (4), 457–72 (1992).
7. A. Gelman et al., Bayesian Data Analysis, 3rd ed., Chapman and Hall/CRC Press, Boca Raton, Fla. (2014).
